# RF performances of inductors integrated on localized p^+^-type porous silicon regions

**DOI:** 10.1186/1556-276X-7-523

**Published:** 2012-09-25

**Authors:** Marie Capelle, Jérôme Billoué, Patrick Poveda, Gaël Gautier

**Affiliations:** 1 Université François Rabelais de Tours, GREMAN, UMR CNRS 7347, 16 Rue Pierre et Marie Curie, BP 7155, Tours, Cedex 2, 37071, France; 2 STMicroelectronics, 16 rue Pierre et Marie Curie, BP 7155, Tours, Cedex 2, 37071, France

**Keywords:** Inductor, Localized porous silicon, Radiofrequency, Quality factor, Resonant frequency, Strain, Warp, Fluoropolymer, Fluorocarbon

## Abstract

To study the influence of localized porous silicon regions on radiofrequency performances of passive devices, inductors were integrated on localized porous silicon regions, full porous silicon sheet, bulk silicon and glass substrates. In this work, a novel strong, resistant fluoropolymer mask is introduced to localize the porous silicon on the silicon wafer. Then, the quality factors and resonant frequencies obtained with the different substrates are presented. A first comparison is done between the performances of inductors integrated on same-thickness localized and full porous silicon sheet layers. The effect of the silicon regions in the decrease of performances of localized porous silicon is discussed. Then, the study shows that the localized porous silicon substrate significantly reduces losses in comparison with high-resistivity silicon or highly doped silicon bulks. These results are promising for the integration of both passive and active devices on the same silicon/porous silicon hybrid substrate.

## Background

The tremendous growth of mobile and wireless applications during the past decade has accelerated the development of radiofrequency (RF) technologies. More and more radio frequency-integrated circuits (RFICs) have been integrated on a single chip, with the advantage of high density of the package, low cost and small volume. To integrate both active and passive devices and to reduce substrate losses, RFIC integration could be made on substrates such as silicon on glass [1], silicon on sapphire or high-resistivity silicon (HR Si) [2,3]. However, CMOS processes generally require low-resistivity silicon substrates, which are lossy and responsible for the deterioration of RF performances [3].

An alternative solution is the use of silicon/porous silicon hybrid substrates. Porous silicon (PS) is known for its insulating properties. Indeed, PS electrical conductivity (*σ*_*PS*_) increases with frequency but remains very low even in the range of a few gigahertz [4]. Ben Chorin and co-workers measured a conductivity modification from 10^−8^ to 10^−5^/Ω cm from direct current (DC) signal to 10 kHz [5]. Balagurov and Timoshenko report mesoporous silicon *σ* of 10^−7^/Ω cm in DC [6,7]. Then, the permittivity of PS (ɛ_PS_) varies between 2 and 11.7 with the porosity according to a Vegard law. Experimental values reported in the literature have already confirmed the decreasing behavior of ɛ_PS_ when the porosity increases [8]. The porous silicon electrical properties allow the reductions of leakage currents and eddy currents in the high-frequency field. Numerous authors show interest in this substrate with regard to bulk silicon for inductor performances (resonant frequency and quality factor) [9]. In previous work, an increase of 250% of the maximum quality factor (*Q* factor) has been calculated between a 100-μm PS layer and a low-resistivity Si substrate [10]. Integration of RF devices on localized porous silicon areas has been addressed by Populaire and Chen [11,12].

Nevertheless, substrate losses with hybrid substrates are increased with regard to full PS sheet layers since lateral couplings with the highly conductive Si are added at the edge of PS regions. That is why deep localized PS layer areas are generally used (>100 μm). Their fabrication requires the use of a strong, resistant mask in HF-based electrolytes for long-time anodizations. Resists or metals different from noble metals have a limited resistance in HF-based solutions [13]. Noble metals are more inert in HF, but charge accumulation at the edges of the openings could be responsible for local high-current density and silicon erosion in these areas. The strong resistance of carbon and silicon carbide layers have been respectively shown by Djenizian [14] and Steiner and Wang [13,15]. Oxide/polysilicon bilayers are also a mask solution [16-18]. However, the common material employed to localize PS is a non-stoechiometric nitrid [15]. The mask removal conditions are also a critical parameter since they have not deteriorated the fabricated PS surface.

In this work, planar inductors integrated on deep localized mesoporous silicon regions and other substrates have been characterized. First, the fabrication processes are detailed. Secondly, the RF performances of the inductors are studied by observing the *Q* factors and resonant frequencies. To judge the impact of the localized PS substrate on RF performances, a comparison is done with full porous silicon sheet and other common substrates such as silicon and glass.

## Methods

### Porous silicon localization

The PS localized regions were defined thanks to a 300-nm-thick fluorine-based (FbF) mask reported by Defforge [19]. It was plasma-deposited at ambient temperature in an inductive coupled plasma equipment at 80 W, with C_2_H_4_ and CHF_3_ as gases precursors. The deposition rate is 35 nm/min. Then, a 500-nm plasma-enhanced chemical vapor deposition (PECVD) oxide was deposited and patterned to be used as a hard mask. The openings in the FbF were performed by etching in an O_2_ plasma, with an etching rate of 360 nm/min. Then, the oxide hard mask was etched by the HF solution during anodization. After the anodization, the FbF was removed with an O_2_ plasma, without damaging the PS region fabricated.

### Anodization settings

**Figure 1:**
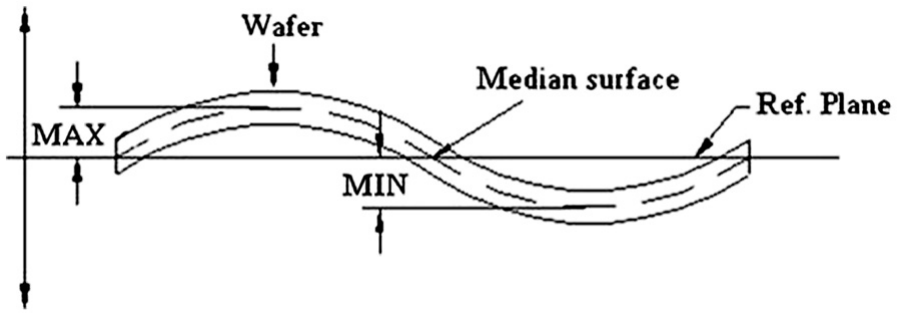
**Schematic view of a wafer warp.** The warp is revealing the curvature of a wafer. It is calculated from a measure of the maximum deviation from the reference plane (MAX) and the minimum deviation from the reference plane (MIN). The warp value is equal to the difference between MAX and MIN.

Both full PS sheet and localized regions were fabricated by anodization of p^+^-doped (111) silicon (*ρ* = 20 mΩ cm) in a double-tank electrochemical cell. The thickness of the Si wafers is 550 μm. The electrolyte used was a HF (50%)/acetic acid/water solution (4.63:2.14:1.43). A current density of 28 mA/cm^2^ was applied, and the corresponding current was calculated according to the surface (opened or total) anodized. First, 100 and 200-μm-thick localized regions were obtained respectively for 123- and 246-min anodization durations. The under-mask etching measured for the 100- and 200-μm PS layers are respectively 80 and 160 μm. Secondly, 100- and 200-μm full PS sheet thicknesses were obtained for 80- and 160-min anodizations. The measured average porosity of the layers is about 50%. At the end of the PS etching process, the substrates have been annealed at 300°C under N_2_ for 1 h in order to stabilize the structure. During the annealing, hydrogen atoms chemisorbed during the anodization are desorbed, which causes the lattice contraction of the PS layer [20,21]. This microscopic effect added with macroscopic stresses, such as porosity gradient in the depth, non-homogeneity of the depth in the whole wafer and oxidation due to annealing, is mainly responsible for the wafer warp (Figure 1). 

**Figure 2:**
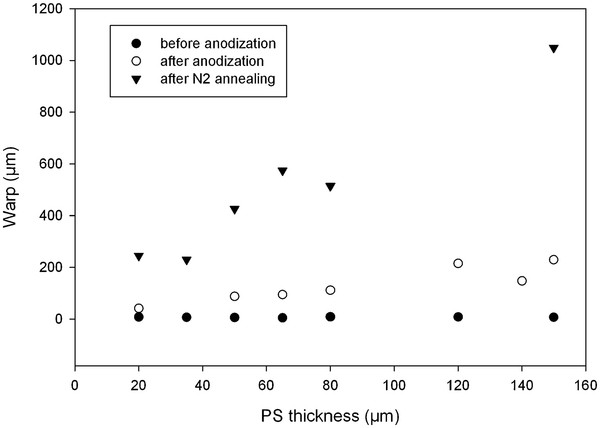
**Evolution of the warp with the PS thickness.** Influence of the PS thickness on the warp of 6-in. p-type silicon (*ρ* = 20 mΩ cm). Measurements were conducted after the anodization and annealing processes. The surface of the substrate was totally anodized. PS layers had average 50% porosity. The annealing was run for 1 h under N_2_ at 300C.

The warp has been measured on full porous silicon sheet layers fabricated on 6-in. p-type (20 mΩ cm) (111) silicon bulk. Figure 2 shows the increase of wafer warp with the PS thickness after a 300C annealing. Generally, a wafer warp superior to 400 μm is responsible for handling issues of non-homogeneity of photolithography and difficulties in aligning mask levels. Since the work was conducted on 25-cm^2^ samples, the warp effect is reduced and no handling issues have been experienced. However, one can make the assumption that the PS localization allows the decrease of warp with regard to full PS sheet.

### Inductor fabrication

**Figure 3:**
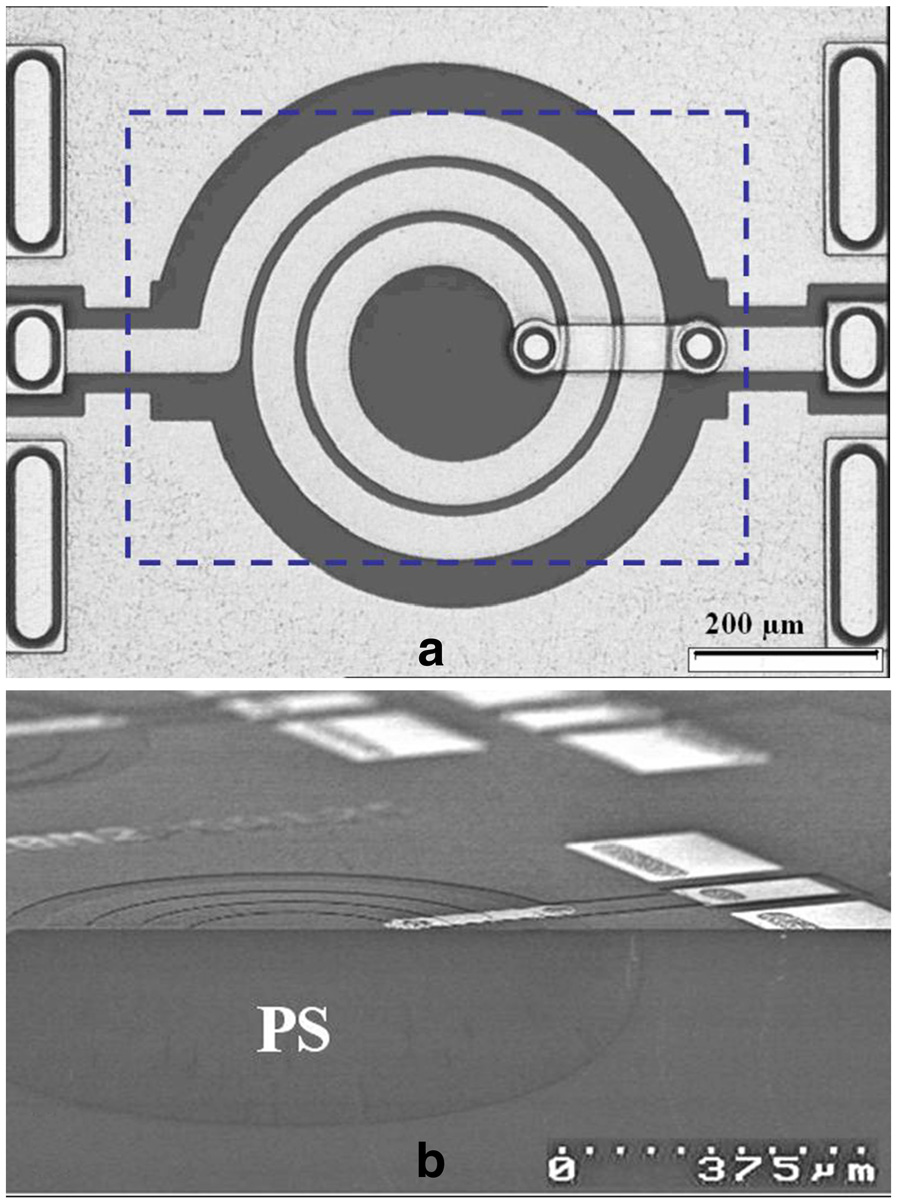
**Planar inductor integrated.** (**a**) Top view of a W50N25R80 planar inductor. The edge of the PS region is defined by the dashed line. (**b**) Tilted cross section of an inductor integrated on the PS region (SEM picture).

Planar inductors were integrated on the PS substrates previously described, on glass, on 3-kΩ cm and 20-mΩ cm Si substrates. The surface of the localized PS region is a rectangle designed under inductor winding (Figure 3). Due to the isotropy of the etching, the PS surface is increased by the PS fabrication under the mask. The ratio between the surface of the inductor metal coil and the PS region is given in Table 1.

First, a 500-nm oxide layer was deposited by PECVD on top of the substrate. The inductor stack is made of two aluminum layers (1 μm) deposited at 350C by physical vapor deposition. The metal layers are separated by a 500-nm PECVD oxide. Patterns have been defined by standard photolithography and dry etching (reactive ion etching).

In this work, 1 to 28 nH inductors with various strip widths (*W*), number of turns (*N*) and internal radius (*R*) were fabricated and characterized. The spacing between adjacent turns (*S*) and the distance to the surrounding ground plane (*S*_g_) are respectively set to 10 and 50 μm. For instance, a 5.5-turn inductor with a 30-μm strip width and an 80-μm internal radius is called W30N55R80.

### RF characterization settings

**Figure 4:**
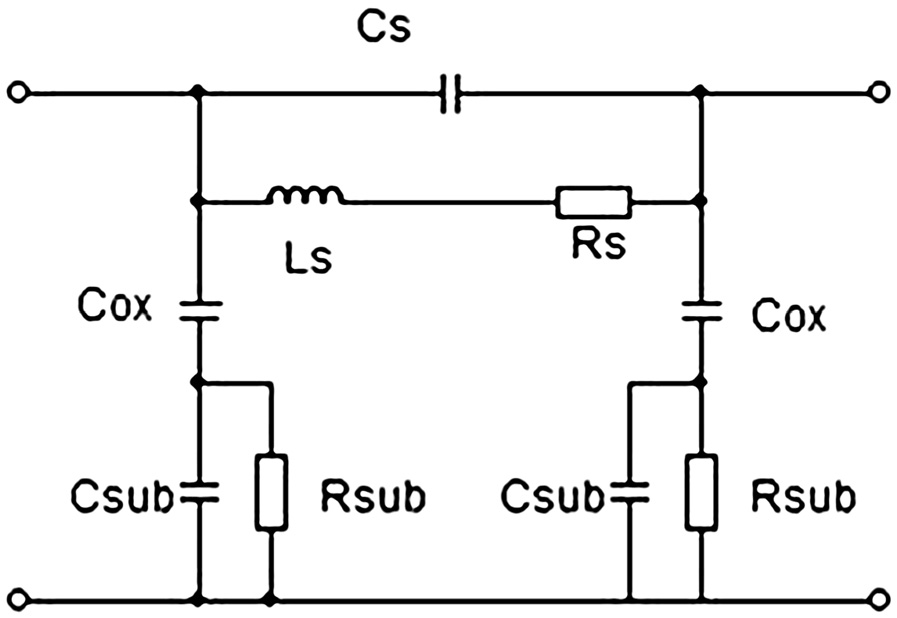
**Electrical equivalent circuit of the inductor.** Typical ‘pi’ model used as an equivalent circuit of the integrated planar inductor. *C*_s_, *L*_s_ and *R*_s_ represent respectively the capacitance, inductance and resistance of the inductor. *C*_ox_ is the capacitance of the oxide. *C*_sub_ and *R*_sub_ are the capacitance and resistance of the substrate.

To compare the performances of the inductors integrated on localized PS with common substrates, *S* parameters were measured using a network analyzer between 10 MHz and 20 GHz. The preliminary calibration was done with the line-reflect-reflect-match method. Then, a conventional three-step de-embedding procedure (using thru, open and short patterns) has been applied to extract the device characteristics from the raw data. To evaluate the substrate losses, the quality factor (*Q*_11_) derived from the admittance (*Y*) matrix when one port is shorted has been calculated (Equation 1). The quality factor is a frequency-dependent parameter, and its value results in energetic electrical losses through the inductor and in the substrate. Thus, higher the Q_11_, better the performances of the inductor are. The inductance *L*_12_ was also calculated and is frequency dependent (Equation 2). Generally, the inductor is used at frequencies below the resonant frequency (*f*_r_), and for the ones, the *L* value is constant. The formula given in Equation 2 is correct only if a pi model is considered (Figure 4). It is an equivalent electrical circuit for both inductor and substrate. Here, the integrated inductor is considered as a two-port network.

(1) Q11=−ImY11ReY11

(2) L12=1ωIm−1Y12

The results of characterization are presented in three parts. A first comparison of inductors’ *Q* factors is done between the localized PS and full PS sheet substrate. Then, it is compared to the results obtained with glass and silicon, which are common substrates used for the integration of RF circuits. To finish, the influence of substrate nature on inductors, resonant frequency (*f*_r_) is presented.

## Results and discussion

### *Q* factor

First, the *Q* factors of the inductors integrated on localized PS and on full PS sheet are compared. Table 1 shows the maximum *Q* factors (*Q*_max_) obtained with these substrates for each inductor design. In any cases, for the same PS thickness, 11% to 46% decreases of the *Q* factor values have been measured between full PS sheet and localized PS. The coverage of the local surface area by the device area is shown by the surface ratio value in Table 1. The expected result would be that the lower the ratio is, the lower is the variation between the *Q* factors of localized and full PS sheet. However, this conclusion cannot be done since surface ratio could be the same for two different inductor designs. The *Q* factor value depends on losses due to the design and substrate. To study properly the effect of the ratio, it will be more rigorous to design several PS region areas and make the comparison on the same inductor design. In this way, losses generated by the design are similar, and the delta-*Q* is directly linked to the surface ratio.

The frequency for one *Q*_max_ obtained is called *f*_Qmax_. Generally, inductors are used at frequencies closed to the *f*_Qmax_ to improve performances. The results show that *f*_Qmax_ obtained with localized PS are lower than the ones with full PS sheet (Table 1).

**Table 1:** ***Q***_**max**_** (*f***_**Qmax**_**) of inductors integrated on localized PS and full PS sheet**

**Inductor design**	**100-μm PS**			**200-μm PS**		
	**Surface ratios**	**Localized PS**	**Full PS sheet**	**Surface ratios**	**Localized PS**	**Full PS sheet**
		***Q***_**max**_** (*f***_**Qmax**_** GHz)**	***Q***_**max**_** (*f***_**Qmax**_** GHz)**		***Q***_**max**_** (*f***_**Qmax**_** GHz)**	***Q***_**max**_** (*f***_**Qmax**_** GHz)**
W10N55R30	0.12	4.7 (2.6)		7.2 (4.7)	0.07	6.9 (3.4)	8.7 (5.4)
W10N55R80	0.13	5.3 (2.3)		6.3 (3)	0.08	-	7.8 (3.2)
W30N15R80	0.12	9.1 (4.2)		12.1 (8.4)	0.07	12.4 (5.3)	22 (12)
W30N35R130	0.22	4.1 (2.2)		6.3 (3.7)	0.15	4.1 (1.9)	7.7 (4)
W50N15R78	0.18	9.4 (3.8)		10.6 (6.6)	0.11	11 (4.6)	17.5 (8)
W50N35R30	0.3	-		6 (2.3)	0.21	4 (1.2)	7.5 (3)
W50N55R175	0.38	3.11 (0.4)		3.8 (0.6)	0.3	-	-

Comparison of the *Q*_max_ obtained with several inductors integrated on localized and full PS sheet substrates. PS layers, 100- and 200-μm thick, are studied. In each case, *Q*_max_ and *f*_Qmax_ are decreased with the PS localization. The surface ratios are the calculated ratios between the surface of the inductor metal coil and the surface of the PS region (the under-mask etching is included in the PS surface). *Q*_max_, maximum *Q* factors; *f*_Qmax_, frequency for the one *Q*_max_.

**Figure 5:**
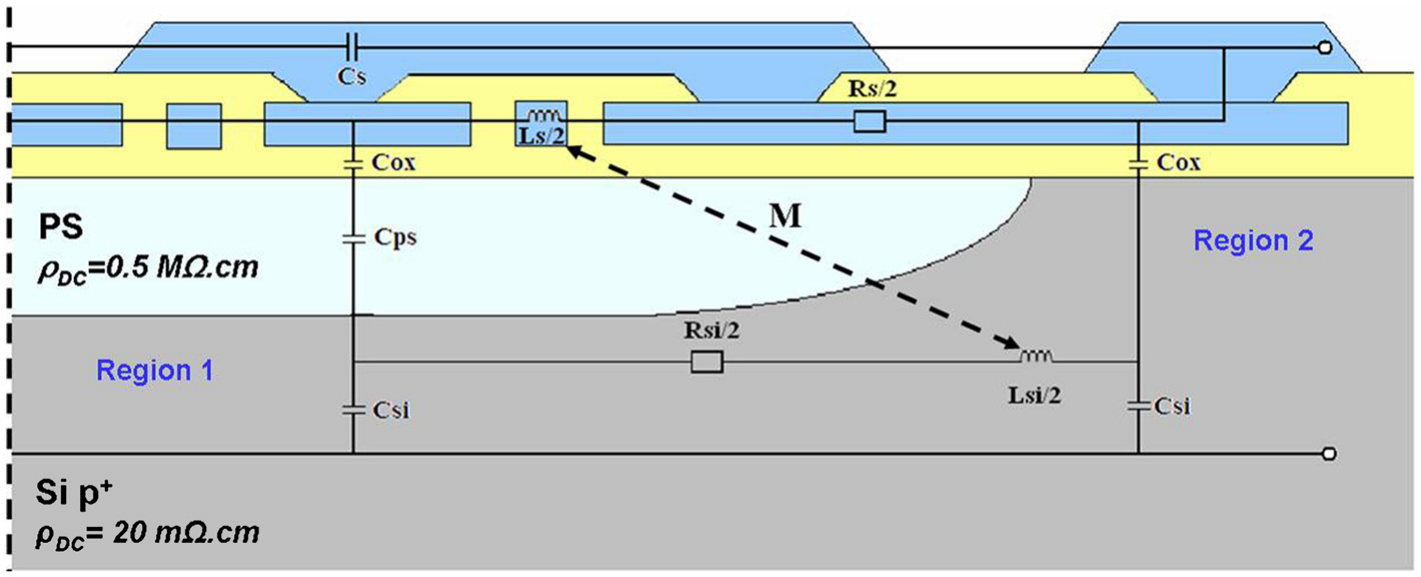
**Electric model of an inductor integrated on localized PS.** Cross-section view of half an inductor (the pad is in the right side). A simplified electric model has been superposed. The aluminum metal coil and oxide layers are represented respectively in blue and yellow. *C*_s_, *R*_s_ and *L*_s_ represent the spiral inductance. *C*_ox_, *C*_ps_ and *C*_si_ are respectively the capacitances of oxide, PS and Si. *R*_si_ and *R*_aps_ are the resistances, and *L*_si_ is the inductance of the two materials. *M* is the mutual inductance between the substrate and the inductor winding.

To understand the effect of the localized PS substrate on the increase of losses, a simplified electric model of an inductor integrated on this substrate is suggested (Figure 5). It is based on Huo’s [22] works, which give several electric models according to substrate resistivity. For this work, the resistivity of the PS regions has been measured, studying the variation of the intensity with voltage. To begin, the spiral inductance is usually represented by the series branch *R*_s_, *L*_s_ and *C*_s_. *L*_s_ is the spiral inductance, and *R*_s_ is the metal series resistance, which is frequency-dependent according to Eddy current generation in the coil. *C*_s_ reflects the capacitance of the winding and between the metal coil level and the underpass. The substrate is represented by *C*_ox_, *C*_ps_, *C*_si_, *R*_si_ and *L*_si_. *C*_ox_ is the oxide capacitance between the winding and the substrate. *C*_ps_ and *C*_si_ are respectively the parallel capacitances of PS and Si. *R*_si_ is the resistance of the high-conductivity Si. The circulation of currents in the inductor metal coils generates magnetic field (*B*). According to the Lenz’s law, the variation of *B* with time induces reverse currents (called Eddy currents) in windings and on the highly doped silicon region. The Eddy current flow in the Si region is responsible for a magnetic field image generation; *L*_si_ is the inductance of the Si substrate. The magnetic coupling with metal coil is indicated by a negative mutual inductance *M*. In addition, this reverse current attracts the main current flowing through the metal coil, which is consequently concentrated on the lower face of the conductor. This proximity effect adds to the conventional skin effect and leads to increased losses. Thanks to the resistivity of the PS region, leakage currents are highly decreased, and it can be assumed that no Eddy currents are generated in this area. 

In the case of the localized PS substrate, the losses caused by the Si bulk below the PS layer (Figure 5, Region 1) are similar to a same-thickness full PS sheet substrate. The hypothesis can be made that additional losses are generated in the silicon area at the edge of the PS region (Figure 5, Region 2) with the localized PS. Some lateral magnetic couplings with the inductor and losses in the pad and conductor pathway may explain the decrease of the *Q*_11_ value.

**Figure 6:**
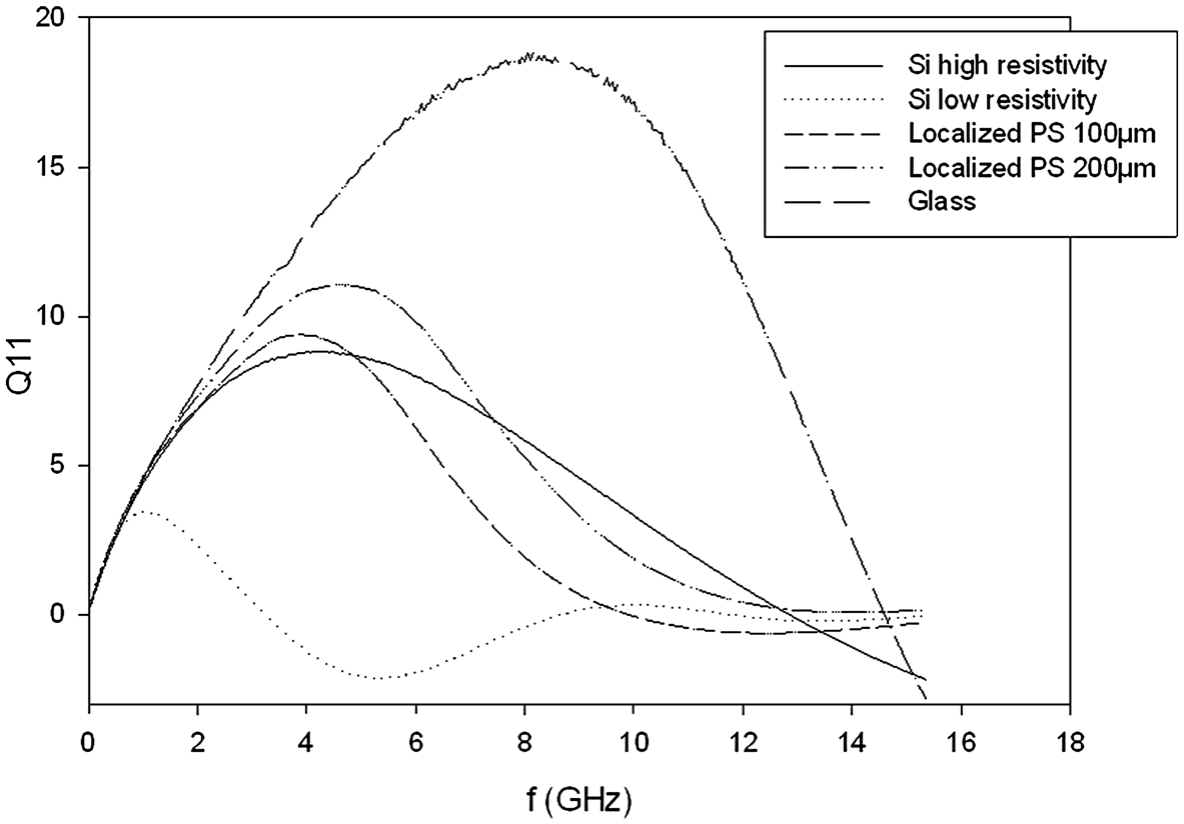
***Q*factor of an inductor integrated on common and PS substrates.** Frequency variation of the *Q* factor of a W50N15R78 inductor integrated on 3-kΩ cm and 20-mΩ cm Si, glass, 100- and 200-μm localized PS substrates. Better *Q*_max_ were obtained with 200- and 100-μm localized PS than with high-resistivity and highly doped silicon substrates.

In a second part, the *Q* factors obtained with inductors integrated on localized PS and other substrates have been studied. Glass and high-resistivity silicon are commonly used for the integration of passive devices for RF applications and provide high *Q* factor inductors. The integration of both active and passive devices is made on highly doped silicon, but mediocre *Q* factors are obtained since electrical losses are high in the substrate. In this study, 550-μm-thick silicon substrates with resistivities of 3 kΩ cm (HR Si) and 20 mΩ cm have been used. Table 2 and Figure 6 show that better *Q*_max_ are obtained with glass than with localized PS. Actually, the relative permittivity (approximately 4) and loss tangent of glass are lower than the PS ones, and substrate losses are reduced. Nevertheless, better *Q*_max_ were obtained with localized PS than highly doped Si. A maximum improvement of 244% of the *Q*_max_ value is measured with 200-μm-thick localized PS. Then, with regard to high-resistivity Si results, better *Q*_max_ have been obtained with 200-μm-thick localized PS. A maximum increase of 84% of the *Q*_max_ value has been observed when comparing both substrates. It can be noticed that the HR Si surface has not been passivated before the oxide deposition, and a MOS capacitance is present at the interface. It results in majority carrier accumulations and generation of additional losses at the surface of the silicon. The solution would be to amorphize the surface by argon implantation or by the deposition of amorphous materials. It has been shown that *Q* factors are improved with the amorphization of the interface [23]. 

**Table 2:** ***Q***_**max**_** (*f***_**Qmax**_**) of inductors integrated on localized PS and other usual substrates**

**Inductor design**	**Q**_**max**_** (*f***_**Qmax**_**) (GHz)**						
	**3-kΩ cm Si**	**20-mΩ cm Si**	**100-μm PS**	**200-μm PS**	**100-μm localized PS**	**200-μm localized PS**	**Glass**
W10N55R30	3.8 (2.6)	1.8 (1)	7.2 (4.7)	8.7 (5.4)	4.7( 2.6)	6.9 (3.4)	8.3 (5.5)
W10N55R80	3.1 (1.6)	1.5 (0.6)	6.3 (3)	7.8 (3.2)	5.3 (2.3)	-	7.8 (3.76)
W30N15R80	9.9 (5.6)	3.6 (1.5)	12.1(8.4)	22 (12)	9.1 (4.2)	12.4 (5.3)	23 (13)
W30N35R130	2.7 (1.7)	1.6 (7.6)	6.3(3.7)	7.7 (4)	4.1 (2.2)	4.1 (1.9)	8.7 (4.5)
W30N55R30	3.5 (9.6)	1.7 (0.3)	5.5 (1.7)	6.6 (2)	4.3 (1.2)	4.5 (1.3)	6.9 (2.15)
W50N15R78	8.8 (4.3)	3.41 (1)	10.6 (6.6)	17.5 (8)	9.4 (3.8)	11 (4.6)	18.7 (7.8)
W50N55R175	2.4 (0.3)	1.2 (0.1)	3.8 (0.6)	-	3.11 (7.1)	-	5.7 (0.9)

Comparison of *Q*_max_ obtained with several inductors integrated on localized PS, full PS sheet and common substrates used for radiofrequency applications. Better *Q*_max_ were obtained with 200-μm localized PS than with highly doped and high-resistivity silicon bulk. PS, porous silicon; *Q*_max_, maximum *Q* factors; *f*_Qmax_, frequency for the one *Q*_max_.

### Resonant frequency

The study of the resonant frequency (*f*_r_) value allows determining the frequency range for which the device behaves like an inductor. This value is determined by studying the evolution of the inductance (*L*_12_) value with the frequency. *f*_r_ is the frequency for which *L*_12_ is equal to 0. The *f*_r_ of several inductors integrated on the substrates described previously have been measured and are summarized in Table 3.

**Table 3:** Resonant frequencies of inductors integrated on PS and other substrates

**Inductor characteristics**		***f***_**r**_**(GHz)**				
**Inductor design**	***L*(nH)**	**Glass**	**3-kΩ cm Si**	**20-mΩ cm Si**	**200-μm full PS sheet**	**100-μm full PS sheet**
W10N55R30	5.3	15.8	15.3	15.6	15.3	-
W50N15R78	1.2	16.7	17	8.3	17	18.2
W50N35R30	3.5	6.5	6.4	4	6.5	7
W50N55R175	21	1.75	1.7	0.7	-	2.1
W10N55R80	10.2	10	9	2	10.5	-
W30N35R130	7	14.5	14	5.11	14.5	-

Resonant frequencies (*f*_r_) of inductors integrated on PS and other substrates. Similar *f*_r_ were obtained with full PS sheet, glass and high-resistivity silicon. *f*_r_ is increased with the PS substrate with regard to highly doped silicon. *f*_r_, resonant frequency.

The values obtained with localized PS have not been published since the pi model (Figure 4) used to calculate the *L*_12_ values does not seem suitable to represent this substrate correctly. It can be confirmed by the negative value of *R*_s_ obtained with localized PS. A suitable model has to be developed to represent the hybrid substrate and extract the correct *L*_12_.

**Figure 7:**
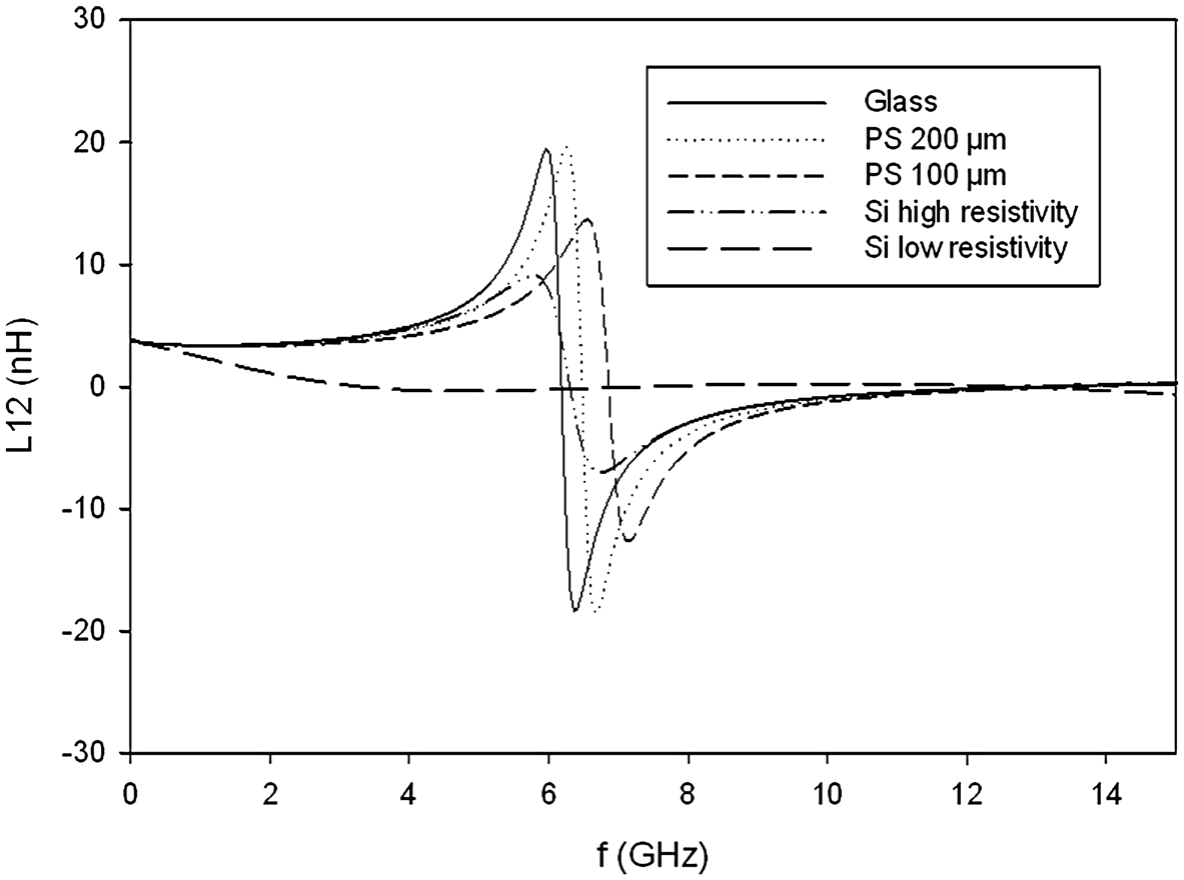
**Resonant frequency measured on common and PS substrates.** Variation of the inductance value (*L*_12_) with frequency for a W50N35R30 inductor integrated on various substrates. Except for highly doped silicon substrates, *f*_r_ between 6 and 7 GHz are obtained.

Concerning other substrates, Table 3 shows the improvement of the *f*_r_ value brought by full porous silicon sheet with regard to highly doped silicon substrate, also measured by Billoué [24]. A maximum improvement of 750% has been calculated with the W10N55R80 inductor. Thus, the inductor can be used at higher frequencies with the integration on PS. Then, the *f*_r_ measured on full PS sheet is equivalent to or higher than that on glass and HR Si. It can be noticed that the inductance values at low frequency are not changed by the PS substrate and are similar from one substrate to another (Figure 7). It confirms that for the same design, the process is reproducible on each substrate. 

## Conclusions

The interest on silicon/porous silicon hybrid substrate for the integration of RF circuits has been studied. To study the influence of hybrid substrate on the performances of passive devices, inductors have been integrated on localized PS and were characterized. The study of the quality factors on localized PS has shown the substrate losses generated by the neighboring highly doped silicon region. However, results are promising since better *Q* factors were obtained with localized PS with regard to highly doped silicon and high-resistivity silicon bulk. Thus, a maximum improvement of 244% of the *Q*_max_ has been obtained with localized PS with regard to highly doped Si bulk. In addition, similar resonant frequencies have been measured with full PS sheet and glass. Thus, the hybrid substrate is a serious candidate for the integration of passive and active devices since it allows increasing the passive device’s performances with regard to commonly used silicon. In addition, performances can still be improved by oxidizing the porous silicon.

## Abbreviations

FbF: fluorine-based film; *f*_Qmax_: frequency for one Q_max_ is reached; *Q*_max_: maximum quality factor; PECVD: plasma-enhanced chemical vapor deposition; PS: porous silicon; RF: radiofrequency; RFIC: radio frequency-integrated circuit.

## Competing interests

The authors declare that they have no competing interests.

## Authors’ contributions

MC fabricated the porous silicon samples and performed the inductor integrations. MC and JB conducted the s-parameter measurements and dealt with the results. MC wrote the article. PP and GG participated in the conception of the study and revised the manuscript. All authors read and approved the final manuscript.
